# Enabling Human Space Exploration Missions Through Progressively Earth Independent Medical Operations (EIMO)

**DOI:** 10.1109/OJEMB.2023.3255513

**Published:** 2023-03-10

**Authors:** Dana R. Levin, Jon Steller, Arian Anderson, Jay Lemery, Benjamin Easter, David C. Hilmers, Kris R. Lehnhardt

**Affiliations:** Department of Emergency MedicineBaylor College of Medicine 3989 Houston TX 77030 USA; Department of Preventive Medicine Division of Aerospace MedicineUniversity of Texas Medical Branch 12338 Galveston TX 77555 USA; Department of Maternal Fetal Medicine and Obstetrics and GynecologyUniversity of California Irvine 8788 Irvine CA 92697 USA; Department of Emergency MedicineSchool of Medicine, University of Colorado Anschutz Medical Campus 129263 Aurora CO 80045 USA; Department of Emergency Medicine, School of MedicineUniversity of Colorado Anschutz Medical Campus 129263 Aurora CO 80045 USA; NASA Johnson Space Center 43834 Houston TX 77058 USA; Department of Internal Medicine and PediatricsBaylor College of Medicine 3989 Houston TX 77030 USA; Center for Space Medicine, Baylor College of Medicine 3989 Houston TX 77030 USA; Department of Emergency MedicineBaylor College of Medicine 3989 Houston TX 77030 USA; Center for Space Medicine, Baylor College of Medicine 3989 Houston TX 77030 USA; NASA Johnson Space Center 43834 Houston TX 77058 USA

**Keywords:** Aerospace medicine, ExMC, Mars, NASA, space medicine

## Abstract

*Goal:* Current Space Medicine operations depend on terrestrial support to manage medical events. As astronauts travel to destinations such as the Moon, Mars, and beyond, distance will substantially limit this support and require increasing medical autonomy from the crew. This paper defines Earth Independent Medical Operations (EIMO) and identifies key elements of a conceptual EIMO system. *Methods:* The NASA Human Research Program Exploration Medical Capability Element held a 2-day conference at Johnson Space Center in Houston, TX with NASA experts representing all aspects of Space Medicine. *Results:* EIMO will be a process enabling progressively resilient deep space exploration systems and crews to reduce risk and increase mission success. Terrestrial assets will continue to provide pre-mission screening, planning, health maintenance, and prevention, while onboard medical care will increasingly be the purview of the crew. *Conclusions:* This paper defines and describes the key components of EIMO.

## I. Introduction

### A. Current Operations and the Challenges of Deep Space

The more than six decades of flight crew experience in low Earth Orbit (LEO) have led to great advances in space medicine knowledge, research, and on-mission medical care. However, the space environment will always be hazardous to humans. Astronauts undergo physiologic changes as they transition into and out of gravity wells which often result in short-lived but uncomfortable medical conditions as well as vascular and neuro-vestibular disturbances that may impair performance on short and mid-term missions [1].

Long-duration missions compound these risks by exposing astronauts to increased radiation, loss of bone mineral density, muscular deconditioning, visual derangements, high carbon dioxide levels, decompression illness, behavioral problems from prolonged isolation and confinement, high task load stresses, and conditions that are yet unknown [2].

To date, all crewed spaceflights, apart from the Apollo program, have launched no farther than LEO. This proximity to Earth provides several advantages such as diminished radiation hazards, expeditious evacuation, and real-time communication [1]. Amenities like the International Space Station (ISS) cupola also help crewmembers maintain a sense of connection with the planet below [2].

The large habitable volume of ISS also facilitates design level countermeasures such as large, sophisticated exercise equipment, crewmember personal compartments, the ability to find time apart from one another as needed, and private space for medical conferences [1], [2], [3].

Medical data are transmitted to the ground for interpretation where a designated flight surgeon and deputy are on call 24 h/7 d to provide medical support. They, in turn, can call upon a network of specialists representing all branches of medicine for real-time support within minutes [4], [5]. The proximity to Earth also allows regular exchange of material from the spacecraft. Air and water samples to be returned to Earth for inspection and any necessary equipment, replacement parts, expired medication, or supplies which have been expended can be delivered on relatively short notice [3], [4], [5], [6], [7], [8].

This robust ground support allows the designated crew medical officers (CMO) to receive only a few days’ worth of medical training before launch, just enough to carry out basic medical tasks under guidance by terrestrial physicians [1], [3], [4]. If a medical event requires more complex intervention, some or all crewmembers can board one of the spacecrafts docked to the station and evacuate to definitive care on Earth in less than 24 hours [8].

Since the medical system only needs to support a stricken crewmember for the time it takes to transfer to a higher level of care, the option to evacuate so rapidly substantially decreases the need for onboard medical capabilities. The ability to replace any expended supplies further decreases the medical system mass and volume by reducing the need for material reserves [1], [9], [10].

These aspects make ISS an ideal platform for evaluating innovative devices such as compact blood analyzers and physiological monitors to support the early stages of EIMO development. Innovations, procedures, and training concepts can be tested and perfected within the safety of the primary ISS medical system. The greater the distance from Earth, the more the current operations strategy is strained and the more reliable EIMO components must be [1], [10].

As humans once again venture beyond LEO, crews will experience more and different operational and environmental challenges. Lunar crewmembers will experience up to a 10 second 2-way communication delay between the time it takes light to travel the distance and latency within communication systems [11]. Additionally, resupply missions will be far less frequent and evacuation back to Earth may take as long as 2 weeks [12]. A physician on Earth can still help with time-sensitive medical decisions but procedural guidance becomes more difficult meaning CMOs will need additional training [13]. The prolonged evacuation time and limited ability to send additional supplies means the onboard medical system must be substantially more robust than on ISS [9].

These challenges increase with missions to destinations further away. Anticipated multi-year Mars missions will travel at least six months and roughly 500 million kilometers in each direction [14]. The 2-way communication delay for a Mars mission may be as long as 40 minutes, with evacuation taking as long as the mission itself. Resupply will be all but impossible. The CMO and medical system will need to make accurate diagnoses and anticipate the questions of specialists on the ground to streamline communications and limit the need for repeated back and forth exchanges. If the CMO becomes the patient, the medical system will need either a similarly highly trained backup CMO or a nearly autonomous ability to support the remaining crewmembers [14], [15], [16], [17], [18].

The onboard resources will become even more constrained as the mass, volume, power, and other resources devoted to the medical system are reduced from ISS standards to meet the vehicle needs of exploration class missions. Vehicle resource allocation to system trades will need to be carefully considered as the ability to resupply vanishes and shipboard supplies become a finite resource [12], [14].

The shelf life of many critical medications is less than the anticipated duration of a Mars mission, a fact made worse by the chemical alterations induced by the radiation, humidity, and high carbon dioxide levels of the space environment. Thus, the choice of pharmaceuticals and their storage containers must also be considered [19].

For these reasons Earth-based medical authority must progressively transition to space-based assets as humans travel progressively farther away from LEO. Terrestrial assets will continue to be paramount in pre-mission screening, planning, maintenance, and prevention. Yet delivering care onboard and responding to unexpected medical events will increasingly become the purview of the crew as distance from Earth turns communication delays and dropouts from the exception to the rule.

### B. Prior Attempts to Define EIMO

Initial space medicine operations focused on planning for specific, well-defined missions. The medical system for Project Mercury was designed to manage minor symptoms during brief stays on orbit and prolonged post landing recovery times. It consisted of a leg bag with resources for a single crewmember, an instruction pamphlet, and the spacecraft's 2-way radio [20]. Apollo operations required a more complex design. The need to handle remote medical monitoring, multiple mission phases, and a wider variety of physiological stressors with prolonged evacuation times required the medical “kit” to be integrated into the vehicle itself. Thus, medical planning began to inform engineering requirements [21], [22].

In the ISS era, the relative permanence of the spacecraft meant the medical system had to support a variety of mission profiles without advance knowledge of the specifics. In other words, the ISS design needed to account for general medical capabilities while retaining the flexibility to add capabilities as mission risk profiles changed [22].

The need to adapt medical system design based on mission risk is described in NASA Standard 3001. The version of this document published in 2014 defined 6 levels of care (0-5) for medical systems based on a heuristically generated, anticipated risk level (see Table I) [23].

**TABLE I:** NASA Standard 3001 Revision 1A Levels of Care

Level of Care	Mission Duration	Heuristic Risk	Routine Care Resourced	Emergency Care Resourced	Level of Independence	Example Mission
0	–	No Risk	None	None	–	T-38 Flight Training
1	“Short”	Limited	None	First Aid Plan for follow on support	Real time comms and evacuation (minutes)	Transfer missions to LEO and Suborbital flights
2	Less than 30 days	Moderate	Clinical Diagnostics Ambulatory Care	Basic Life Support	Real time comms and evacuation (hours)	LEO
3	Less than 30 days	Moderate to high	Clinical Diagnostics Ambulatory Care Limited Dental care	Advanced Life Support Trauma Care	Real time comms and evacuation (hours)	Lunar, Planetary
4	30-210 days	Moderate to high	Clinical Diagnostics Ambulatory Care Increased Dental care	Increased Advanced Life Support Increased Trauma Care	Real time comms Prolonged Evacuation (days)	Lunar, Planetary, Long LEO
5	Greater than 210 days	High	Autonomous Advanced Care	Autonomous Advanced Life Support Increased Trauma Care	Comms limited, Evacuation not an option	Lunar, Planetary
Table adapted from the text of NASA Standard 3001 Revision 1A [23]

**TABLE II:** Important Domains of EIMO Pre Mission Planning

Domain	Description
1.	Define the anticipated activities
2.	Identify the relevant, treatable conditions
3.	Define mission relevant outcomes (ex. task time lost, loss of crew life, need for evacuation)
4.	Collect data to support PRA
5.	Set acceptable risk tolerance level
6.	Run PRA
7.	Adapt PRA output for specific mission operations
8.	Set system requirements
9.	Coordinate with engineers to ensure requirements are fulfilled
10.	Determine the required onboard Knowledge, Skills, and Abilities (KSA)
11.	Determine required skills for each crewmember
12.	Design detailed and Just-in-Time pre-flight trainings to train crewmembers to desired KSA levels
13.	Design appropriate onboard routine training/practice modules
14.	Design onboard Just-in-Time Training (JITT)
15.	Establish mission support schedules
16.	Establish mission support protocols and flight rules

The highest level of care, level 5, applied to hypothetical Lunar and planetary missions lasting longer than 210 days. The document described a high risk for medical events on these missions and specified that crew caregivers must be trained to the physician level. Since evacuation to Earth may not be possible, these missions were described as requiring a high level of autonomy and a policy for terminating care in cases of medical futility or when continued care of a sick crewmember may put healthy crew at risk. The document does not provide full specifics on these topics but was intended to guide mission planners as they began to consider what these specifics might be [23].

NASA Standard 3001 Revision 1A is one of the first official attempts to define EIMO. However, the definition in this version is rigid and tied to specific mission profiles, imposing unnecessary restrictions on mission planners without considering a progressive approach. For example, it primarily defines risk by duration rather than distance and applies the same requirements to both Lunar and Martian missions despite the substantial difference in communication, evacuation, and resupply abilities.

Since distance from Earth in addition to duration will drive mission risk and the planned missions may be different than the profiles outlined in the standard, the risk levels defined in the document may not apply to anticipated missions.

To address this NASA Standard 3001 was revised in 2022 to replace the level of care definitions with a requirement to define medical system requirements by mission profile specific Probabilistic Risk Assessment (PRA) [24]. PRA is a computational tool that uses evidence-based adverse outcome occurrence rates to equate risk across disparate events [25].

Medical PRA calculates the resource cost of treating medical conditions based on variable mission parameters like crew size, duration, mass/volume/power allocation, and specific mission tasks [26]. It does this by drawing from a database of condition incidence, treated/untreated outcomes and diagnostic/treatment resourcing for each medical condition anticipated on the mission profile. These data are run through a computational model that simulates thousands of missions and produces an estimate of how often medical conditions may occur and the risk difference between treating or not treating them. An algorithm uses these estimates to “design” the optimal medical system for reducing risk based on the desired mission profile outcome and the mission resource cost (mass, volume, power use, etc.) associated with that benefit. This ties medical risk planning to evidence-based estimates and allows planners to translate medical conditions and treatment into engineering relevant costs [26], [27].

PRA is a powerful planning tool, but it is still dependent on experts populating the database with appropriate medical capabilities and resources, and future mission risk reductions depend on technology, procedure, and training innovation. For these reasons a clear, updated definition for EIMO has become a high priority.

## II. Materials and Methods

To establish a definition for EIMO and develop a high-level outline of EIMO system components, a team of 30 NASA affiliated experts in space medical operations and space medical systems design was assembled by NASA's Human Research Program and Exploration Medical Capability Element (ExMC). This team included physicians, systems engineers, former flight controllers, computational modelers, human factors engineers, software engineers, technical managers, and a physician astronaut.

Physician expertise included board certifications in aerospace medicine, emergency medicine, internal medicine, family medicine, sports medicine, obstetrics and gynecology, and critical care. Additional formal training and experience included hyperbaric medicine, wilderness medicine, public health, spacecraft systems, expedition planning, space medicine operations, space medicine research/design, and computational risk modelling. Input was also sought from experts with spaceflight experience, and experts presently working on deep space medical risk assessment and system design.

The team met over two days in June 2022 to review present day space medicine operations, past attempts to define and guide development of EIMO, and NASA design reference missions (DRMs) for long-duration Lunar missions and Martian exploration. The key environmental, operational, and medical challenges of EIMO were identified and system elements to address them were proposed. These challenges and potential solutions were used to derive a proposed definition for EIMO and an outline of EIMO system components.

## III. Results

### A. Updated Definition of EIMO

EIMO is the transition of medical primacy from terrestrial to space-based assets to enable support of astronaut health and performance. This will be a process enabling progressively resilient deep space exploration systems and crews to reduce risk and increase mission success. Terrestrial assets will continue to provide pre-mission screening, planning, maintenance, and prevention while onboard care, response to unexpected medical events, management of communication delays, and caring for crew members during communication dropouts will increasingly be required of the crew.

### B. Potential Components of an EIMO System

#### 1) Pre-Mission Planning

The history of terrestrial and space exploration strongly supports the value of extensive pre-mission planning [21], [27]. Based on the Concept of Operations documents for the planned Gateway and Mars missions and present-day mission operations it is possible to determine several high-yield domains of pre-mission EIMO planning (see Table I) [11], [13].

The first four steps construct a medical Probabilistic Risk Assessment (PRA) model that informs the resourcing, trade-space analysis, and crew training needs. The more robust the prediction and characterization of medical condition occurrence, the more effective the mitigation of adverse outcomes will be.

NASA already relies on PRA to inform medical planning through the Integrated Medical Model (IMM). However, IMM is limited in its ability to support mission planning beyond LEO [26]. IMM's successor, Informing Mission Planning via Analysis of Complex Trade-spaces (IMPACT), aims to address IMM's limitations and enable similar robust planning for the clinical and engineering needs of exploration class missions. Both tools automate key steps in the mission planning checklist to streamline assessment and minimize mission risk. Such systems can also estimate crew training requirements, guide research priorities, and help triage remaining in-flight resources as a mission progresses [27].

#### 2) Acute and Emergent Management Decision Making

Distance, communication latency, and reduced data channel capacity will delay ground-based assistance and eliminate expeditious evacuation [8], [11], [12], [13]. For some conditions, delays in management advice could result in loss of crew life or irreversible disabilities. Thus, onboard caregivers must have sufficient knowledge, skills, and abilities (KSA) to stabilize and manage any anticipated condition long enough to receive definitive care advice from terrestrial experts [28], [29].

These experts must maintain their high level of KSA despite infrequent practice or exposure to emergency medical events. The use of practice sessions, knowledge reviews, and just in time training (JITT) can assist with this [15].

Additionally, computer-based medical decision support tools can help increase the level of onboard KSA through autonomous diagnostic and treatment algorithms that can assist the onboard care providers [17].

#### 3) Prolonged Medical Management Decision Making

Just as data transmission time limits emergency medical care, data channel capacity will likely limit non-emergent care [14]. Significant response latency and low transmission rates in current deep space communication systems affect the ability to transmit high resolution images, video, and other telemedical data in medically meaningful time frames [10]. Thus, even non-emergent conditions may require high onboard KSA.

#### 4) Supplies and Resource Management

Medical resourcing has historically been allocated via expert opinion. Any supplies/use mismatches were either managed by the short duration of the mission or by sending additional supplies into LEO [22].

Exploration class missions cannot rely on resupply, and in some cases, medications may expire or suffer reduced efficacy from environmental stressors before a mission is completed. This means supplies must be carefully chosen to account for mass, volume, stability, and re-usability [19].

PRA tools such as IMM and IMPACT have tremendous potential to optimize EIMO planning by combining estimations of medical event outcomes with the mass and volume of the medical capabilities needed to treat these conditions. Specially designed algorithms can identify the optimum risk reduction achievable within the mass and volume constraints of a given spacecraft and help mission planners select only the most impactful medical system components [26], [27].

Innovations in regenerable, recyclable, and on-demand resourcing such as 3D printers or plant-based pharmaceutical “farms” will enhance the resilience of exploration medical systems even further. Such mechanisms could ensure that consumable or expirable medical system components can be replaced even when a spacecraft is beyond the reach of resupply missions.

#### 5) Task Load Management

LEO operations allow crewmembers to offload many medical decisions, monitoring, and other tasks to terrestrial experts. This allows a CMO to focus on patient care, avoid task saturation, and reduce stress [4].

EIMO will require CMOs to perform these functions largely autonomously, substantially increasing the cognitive load. This has been shown to impair decision making, affect the quality of patient care, and consequently increase mission risk [30].

One solution is to delegate tasks to computerized assistants. Digital clinical decision support systems (CDSS) can assist with inventory management, locating resources, storage, and retrieval of medical records, highlighting trends in recorded data, scheduling, reminders, and record keeping [18], [30], [31].

Such tools are already integrated into many terrestrial electronic medical records for similar reasons [30], [31]. However, a system designed for EIMO must be more robust, more capable, and more streamlined. EIMO CDSS design must be intuitive to use, reliable, and fast. It should perform all terrestrial CDSS functions, passively monitor the crew for early signs of behavioral or medical anomalies, provide diagnostic assistance, autonomously suggest treatments, and guide crewmembers through procedures. A comprehensive CDSS could enhance many aspects of care that are currently provided by mission control and be the cornerstone of effective EIMO [18].

## IV. Discussion

This new definition for EIMO is intended to establish common ground to begin discussions with the broader space medicine community and facilitate future medical system planning and development. The definition and the components outlined here are not an end point in themselves but rather a progressive concept built on historical lessons.

While each previous program unquestionably informed the next, few would argue that Mercury era support is sufficient for today's ISS. Just as medical support for Mercury and Gemini taught valuable lessons to Apollo, which in turn informed Skylab, Shuttle, Mir-Shuttle, and the International Space Station (ISS), so too will lessons from ISS inform EIMO.

The efforts of this paper are limited to a high-level description based on the knowledge of present NASA space medicine experts. There is much work to be done to further define EIMO and fill in the details of EIMO systems.

It is our hope that the definition and EIMO system components presented in this paper will facilitate and inspire further discussion among NASA, national, international, and commercial partners. These partnerships and continued discussions will help transition space medical systems from present day ISS operations through successful Lunar and Martian missions, eventually enabling full terrestrial standards of care away from our home world.

## V. Conclusion

EIMO will require both terrestrial and space-based components. Pre-mission planning, system design, crew health maintenance, training, and JITT development are best accomplished terrestrially. Similarly, specialist consults, support for in mission medical events and event/resource tracking to enable updated PRA are most efficiently accomplished on Earth. However, the response to non-routine medical events will require levels of onboard decision support, data stream insight, and KSA that are not currently implemented or available, necessitating the development of novel technologies, training, protocols, and support structures built upon the successful strategies and innovations of the past six decades.

## References

[1] T. A. Taddeo , “Spaceflight medical systems,” in Principles of Clinical Medicine for Space Flight, 2nd ed. New York, NY, USA: Springer, 2019, Ch. 6, pp. 206–229.

[2] E. S. Baker , “Human response to spaceflight,” in Principles of Clinical Medicine for Space Flight, 2nd ed. New York, NY, USA: Springer, 2019, Ch. 12, pp. 367–411.

[3] K. Bacal , “A concept of operations for contingency medical care on the international space station,” Mil. Med., vol. 169, no. 8, pp. 631–641, Aug. 2004. 15379076 10.7205/milmed.169.8.631

[4] S. Melton , “Telemedicine,” in Principles of Clinical Medicine for Space Flight, 2nd ed. New York, NY, USA: Springer, 2019, Ch. 8, pp. 253–271.

[5] J. M. Duncan , “Organization and management of the international space station (ISS) multilateral medical operations,” Acta Astronautica, vol. 63, no. 7–10, pp. 1137–1147, Oct. 2008, doi: 10.1016/j.actaastro.2007.12.001.

[6] G. Beck , “Hypoxia, hypercarbia, and atmospheric control,” in Principles of Clinical Medicine for Space Flight, 2nd ed. New York, NY, USA: Springer, 2019, pp. 140–141.

[7] W. T. Wallace , “Monitoring of the atmosphere on the international space station with the air quality monitor,” in Proc. 47th Int. Conf. Environ. Syst., Charelston, SC, USA, 2017, Paper ICES-2017-103. [Online]. Available: https://ntrs.nasa.gov/citations/20170002579

[8] T. F. Limero , “What air and water quality monitoring is needed to protect crew health on spacecraft?,” New Space, vol. 5, no. 2, pp. 67–78, Jun. 2017. [Online]. Available: https://www.liebertpub.com/doi/full/10.1089/space.2017.0004

[9] S. L. Johnston III , “Medical evacuation risk and crew transport,” in Principles of Clinical Medicine for Space Flight, 2nd ed. New York, NY, USA: Springer, 2019, Ch. 10, pp. 201–231.

[10] D. Risin, “Risk of inability to adequately treat an ill or injured crew member,” in Human Health and Performance Risks of Space Exploration Missions, J. C. Mcphee and J. B. Charles, Eds. Houston, TX, USA: NASA, 2009, pp. 239–250. [Online.] Available: [Online]. Available: https://humanresearchroadmap.nasa.gov/Evidence/reports/EvidenceBook.pdf

[11] E. Kenny , “Human flight to lunar and beyond – re-learning operations paradigms,” in Proc. SpaceOps Conf., 2016. [Online]. Available: https://ntrs.nasa.gov/api/citations/20160004938/downloads/20160004938.pdf

[12] D. Rubin , “Recommendation for a medical system concept of operations for gateway missions,” presented at NASA HRP Investigators Workshop, Galveston, TX, USA, Jan. 27-30, 2020, Paper 20200001341. [Online]. Available: https://ntrs.nasa.gov/citations/20200001341

[13] T. H. Kamine , “Impact of time delay on simulated operative video telementoring: A pilot study,” Aviation Space Environ. Med., vol. 93, no. 2, pp. 123–127, Feb. 2022. 10.3357/AMHP.5972.2022 35105431

[14] M. Urbina , “Medical system concept of operations for mars exploration mission,” presented at NASA HRP Investigators Workshop, Galveston, TX, USA, Jan. 22-25, 2019, Paper 20200001715. [Online]. Available: https://ntrs.nasa.gov/citations/20200001715

[15] M. Krihak , “Communication bandwidth considerations for exploration medical care during space missions,” NASA. Moffet Field, CA., Aug. 2019. [Online]. Available: https://ntrs.nasa.gov/citations/20200001702

[16] R. S. Blue , “Identification of medical training methods for exploration missions,” NASA. Alexandria, VA. Jan. 2014. [Online]. Available: https://ntrs.nasa.gov/api/citations/20130013532/downloads/20130013532_Final.pdf

[17] E. Antonsen, “Human system risk in spaceflight presentation for NASA human research program exploration medical capability element,” NASA. Houston, TX, Jul. 2019. [Online]. Available: https://ntrs.nasa.gov/api/citations/20190032988/downloads/20190032988.pdf

[18] B. L. Beard , “Supporting crew medical decisions on deep space missions : A real-time performance monitoring capability,” presented at the Int. Assoc. Advance. Space Saf. Conf., Rotterdam, The Netherlands, Oct. 19-21, 2021.

[19] R. S. Blue , “Supplying a pharmacy for NASA exploration spaceflight: Challenges and current understanding,” NPJ Microgr., vol. 5, Jun. 2019, Art. no. 14, doi: 10.1038/s41526-019-0075-2. PMC6565689 31231676

[20] M. M. Link, “Mercury medical operations,” Space Medicine in Project Mercury, Washington, DC, USA: NASA, 1965, pp. 135–168. [Online]. Available: https://history.nasa.gov/SP-4003.pdf

[21] C. A. Berry , “History of space medicine: The formative years at NASA,” Aviation, Space, Environ. Med., vol. 80, no. 4, pp. 345–352, Apr. 2009. 10.3357/asem.2463.2009 19378903

[22] K. Fong, “Moon landing: Space medicine and the legacy of Project Apollo,” Lancet, vol. 394, no. 10194, pp. 205–207, Jul. 2019. 31327360 10.1016/S0140-6736(19)31568-5

[23] NASA Office of the Chief Health and Medical Officer, NASA-STD-3001, Space Flight Human-System Standard Volume 1 Revision A: Crew Health, NASA-Standard-3001 VOL 1, NASA. Washington, D.C., Jul. 2014. [Online]. Available: https://standards.nasa.gov/sites/default/files/standards/NASA/B/Historical/2014_07-30_NASA-STD-3001-Vol-1-Rev-A_published.pdf

[24] NASA Office of the Chief Health and Medical Officer, NASA-STD-3001, Space Flight Human-System Standard Volume 1 Revision B: Crew Health, NASA-Standard-3001 VOL 1, NASA. Washington, D.C., Jan. 2022. [Online]. Available: https://standards.nasa.gov/standard/NASA/NASA-STD-3001-VOL-1

[25] M. Stamatelatos , “Probabilistic risk assessment procedures guide for NASA managers and practitioners,” NASA. Washington, D.C., Tech. Rep. HQ-STI-11-213, Dec. 2011. [Online]. Available: https://ntrs.nasa.gov/citations/20120001369

[26] E. L. Kerstman , “The integrated medical model: A risk assessment and decision support tool for human space flight missions,” presented at the 82nd Sci. Meeting Aerosp. Med. Assoc., Anchorage, AK, USA, May 8-12, 2011. [Online]. Available: https://ntrs.nasa.gov/citations/20100036908

[27] E. L. Antonsen , “Estimating medical risk in human space flight,” NPJ Microgr., vol. 8, no. 8, pp. 1–8, 2022, doi: 10.1038/s41526-022-00193-9. PMC8971481 35361776

[28] R. E. Suter, “Emergency medicine in the United States: A systemic review,” World J. Emerg. Med., vol. 3 no. 1, pp. 5–10, Feb. 2012. 25215031 10.5847/wjem.j.issn.1920-8642.2012.01.001 PMC4129827

[29] P. J. McGinnis , “The re-emergence of space medicine as a distinct discipline,” Aviation, Space, Environ. Med., vol. 69, no. 11, pp. 1107–1011, Nov. 1998. 9819171

[30] J. Varghese , “Effects of computerized decision support system implementations on patient outcomes in inpatient care: A systematic review,” J. Amer. Med. Inform. Assoc., vol. 25, no. 5, pp. 593–602, Sep. 2018, doi: 10.1093/jamia/ocx100. 29036406 PMC7646949

[31] R. T. Sutton, “An overview of clinical decision support systems: Benefits, risks, and strategies for success,” NPJ Digit. Med., vol. 3, 2020, Art. no. 17. [Online]. Available: https://www.nature.com/articles/s41746-020-0221-y 10.1038/s41746-020-0221-y PMC7005290 32047862

